# Predictive Value of Pleural Cytology in the Diagnosis of Complicated Parapneumonic Effusions and Empyema Thoracis

**DOI:** 10.1155/2020/7175451

**Published:** 2020-05-20

**Authors:** John Ferguson, Michal Kazimir, Michael Gailey, Frank Moore, Earl Schott

**Affiliations:** ^1^Rocky Mountain Pulmonary and Critical Care, 3555 Lutheran Pkwy, Suite 150. Wheat Ridge, CO 80033, USA; ^2^Midtown Inpatient Medicine, 835 E. 18th Ave. Denver, CO 80218, USA; ^3^Ameripath-Denver. 695 S, Broadway, Denver, CO 80209, USA; ^4^Rocky Mountain Radiologists, 1873 S. Bellaire St, #420. Denver, CO 80222, USA

## Abstract

**Introduction:**

Complicated parapneumonic effusions (CPE) are distinguished from uncomplicated parapneumonic effusions (UPE) by the ability to resolve without drainage. Determinants include pleural pH, pleural glucose, and pleural LDH, along with microbiologic cultures. Inflammation mediated by neutrophil chemotactic cytokines leads to fibrinous loculation of an effusion, and the degree of this inflammation may lead to a CPE. One role of the pathologist is to evaluate for the presence of malignancy in a pleural effusion; however, the ability of the pathologist to distinguish a CPE from UPE has not been evaluated.

**Materials and Methods:**

A single-center retrospective study was performed on pleural cytology specimens from 137 patients diagnosed with a parapneumonic effusion or empyema over a five-year interval. Pleural cytology was characterized as either uncomplicated or complicated by two pathologists based on cellular composition and the presence or absence of fibrinous exudate in the fluid. Cohen's kappa was calculated for interobserver agreement. The sensitivity, specificity, positive predictive value (PPV), and negative predictive value (NPV) of cytologic diagnoses were calculated. Determinants of cytologic accuracy were assessed using Wilcoxon rank sum test, unpaired *t*-test, and logistic regression.

**Results:**

Kappa interobserver agreement between pathologists was 0.753. Pleural fluid cytology sensitivity, specificity, PPV, and NPV for CPE/empyema were 76.0%, 95% CI [65.0, 84.9]; 50%, 95% CI [29.1, 70.9]; 83.3%, 95% CI [76.7, 88.4]; and 38.7%, 95% CI [26.5, 52.5], respectively. The presence of pleural bacteria, elevated pleural LDH, and reduced pleural pH were nonsignificant determinants of cytologic accuracy. Logistic regression was significant for the presence of pleural bacteria (*p* = 0.03) in determining a successful cytologic diagnosis.

**Conclusion:**

Pleural cytology adds little value to traditional markers of distinguishing a UPE from CPE. Inflammation on pleural fluid cytology is suggestive of empyema or the presence of pleural fluid bacteria.

## 1. Introduction

Parapneumonic effusions are commonly encountered complications of community-acquired pneumonia. As many as 40% of patients hospitalized with a diagnosis of pneumonia develop a parapneumonic effusion, and 5-10% of parapneumonic effusions progress to empyema [1]. A complicated parapneumonic effusion (CPE), by definition, requires drainage for infection resolution, whereas uncomplicated parapneumonic effusions (UPE) will resolve with antibiotics alone.

Clinical criteria used to differentiate a CPE from a UPE include pleural glucose less than 40 mg/dL, pleural LDH greater than 1000 IU/mL, pleural pH less than 7.2, the presence of pus (empyema), or positive pleural bacteria culture. However, these criteria are based on the characteristics of patients for whom tube thoracostomy is often performed in clinical practice [2]. Notably, many effusions that are considered complicated by these above listed clinical criteria will resolve with antibiotics and time alone [3]. A gold standard for indications of pleural space drainage is lacking.

The majority of parapneumonic pleural effusions are exudative with polymorphonuclear leukocytes (PMNs) [4, 5]. The presence of PMNs in the pleural fluid is indicative of acute pleural inflammation, often due to bacterial pneumonia [4–6]. Pleural fluid IL-1-beta [7], IL-8 [8, 9], and monocyte chemotactic peptide-1 (MCP-1) are increased in parapneumonic effusions. These cytokines are chemotactic for neutrophils and monocytes in empyema pathogenesis [8, 9]. Increased levels of IL-8, neutrophil elastase, and myeloperoxidase lead to acute pleural inflammation in empyema [10]. Also, TNF-alpha levels correlate inversely with pH in patients with CPE, and IL-1, IL-6, IL-8, and macrophage inflammatory protein-1B (MIP) are significantly higher than in UPE [11–13]. Loculated parapneumonic effusions demonstrate reduced fibrinolytic activity, increased TNF-alpha, IL-1, and TGF [14].

We hypothesize that pleural fluid inflammation seen on pleural fluid cytology is predictive of a diagnosis of a CPE.

## 2. Materials and Methods

### 2.1. Patient Selection

One hundred thirty-seven patients who were treated for parapneumonic effusion or empyema from January, 2014, to January, 2019, were retrospectively identified. Institutional review board approval was obtained at the treating institution.

Pleural fluid clinical characteristics were used to differentiate a CPE from UPE. A CPE was defined as a pleural effusion with glucose less than 40 mg/dL, LDH greater than 1000 U/mL, pH less than 7.2, gram stain positive, or culture positive. A UPE was defined as a PPE meeting none of these criteria. Empyema (frank pus) and CPEs were combined into a single category. A subsequent evaluation of cytology performance was employed using pH, LDH, and glucose criteria as the standard with a positive culture or empyema as a resolver.

Cytology smears were prepared from pleural fluid by a standard liquid-based cytology method (ThinPrep, Hologic) and stained with Papanicolaou stain and by the cytospin technique and stained with Wright-Giemsa stain. Characteristics of the smears included relative cellularity, predominance of cell type (neutrophil, macrophage, and lymphocyte), presence or absence of fibrinous exudate, presence or absence of hemocontamination, and presence or absence of paucicellular, proteinaceous fluid. A determination was made by the pathologist of UPE or CPE based on these characteristics. A pilot analysis of 10 cases was reviewed by Pathologist A. Nine cytologic diagnoses were concordant with the clinical diagnosis of UPE or CPE/empyema in this evaluation.

Pleural fluid samples from these study patients were reviewed independently by two pathologists. Each pathologist characterized by cytologic appearance whether the effusion was uncomplicated or complicated/empyema. In the event of discordant interpretation between pathologists, a third joint pathology review designated the effusions as complicated or uncomplicated to reach consensus.

### 2.2. Outcomes

The primary outcomes were the positive and negative predictive values of cytological prediction of complicated effusions when compared to clinical markers. Secondary outcomes were the interobserver agreement of CPE/empyema as well as predictors of accurate cytological prediction.

### 2.3. Data Collection and Protocol

Study patient charts were reviewed for age, gender, the RAPID score, pleural chemistry (glucose, LDH, pH, gram stain, and culture), presence of pus in pleural fluid, and white blood cell count. The percentage of hemithorax opacity was measured by the admission chest X-ray.

### 2.4. Statistical Analysis

Statistics were performed using Stata version 15.1. The null hypothesis for all comparisons was of no difference in outcomes. A two-tailed alpha of 0.05 was specified for statistical significance. Cohen's Kappa was used to measure interobserver agreement between reviewers. The Shapiro-Wilk normality test was performed on categorical variables. Normally distributed categorical variable outcomes were evaluated using an unpaired *t*-test. Nonnormally distributed variable outcomes were evaluated using a rank sum test. Pleural LDH, pleural glucose, pleural pH, presence of pleural bacteria, pleural pus, and cytologic diagnosis were used to form a logistic regression with clinical-cytologic agreement with the dependent outcome.

## 3. Results

### 3.1. Interobserver Agreement

One hundred thirty-seven pleural fluid cytology specimens were reviewed. The mean age of the population was 64.1 years (16.2 years), and 63.5% were male. Interobserver agreement was reached in 123 specimens (89.8%) and was discordant in 14 (10.2%) (Table 1). Cohen's Kappa (*ϰ*) interobserver agreement was 0.753, 95% CI [0.632, 0.875].

### 3.2. Sensitivity, Specificity, Positive Predictive Value, and Negative Predictive Value

The sensitivity, specificity, PPV, and NPV of Pathologist A for determining CPE/empyema were 76.3%, 95% CI [65.2, 85.3]; 31.2%, 95% CI [19.9, 44.3]; 58%, 95% CI [52.8, 63.0]; and 51.4%, 95% CI [37.9, 64.70], respectively.

The sensitivity, specificity, PPV, and NPV of Pathologist B for determining CPE/empyema were 72.2%, 95% CI [60.4, 82.1]; 36.9%, 95% CI [25.3, 49.8]; 55.9%, 95% CI [50.1, 61.6]; and 54.6%, 95% CI [42.4, 66.2], respectively.

The consensus diagnosis sensitivity, specificity, PPV, and NPV for CPE/empyema were 76.0%, 95% CI [65.0, 84.9]; 50%, 95% CI [29.1, 70.9]; 83.3%, 95% CI [76.7, 88.4]; and 38.7%, 95% CI [26.5, 52.5], respectively.

**Figure 1:**
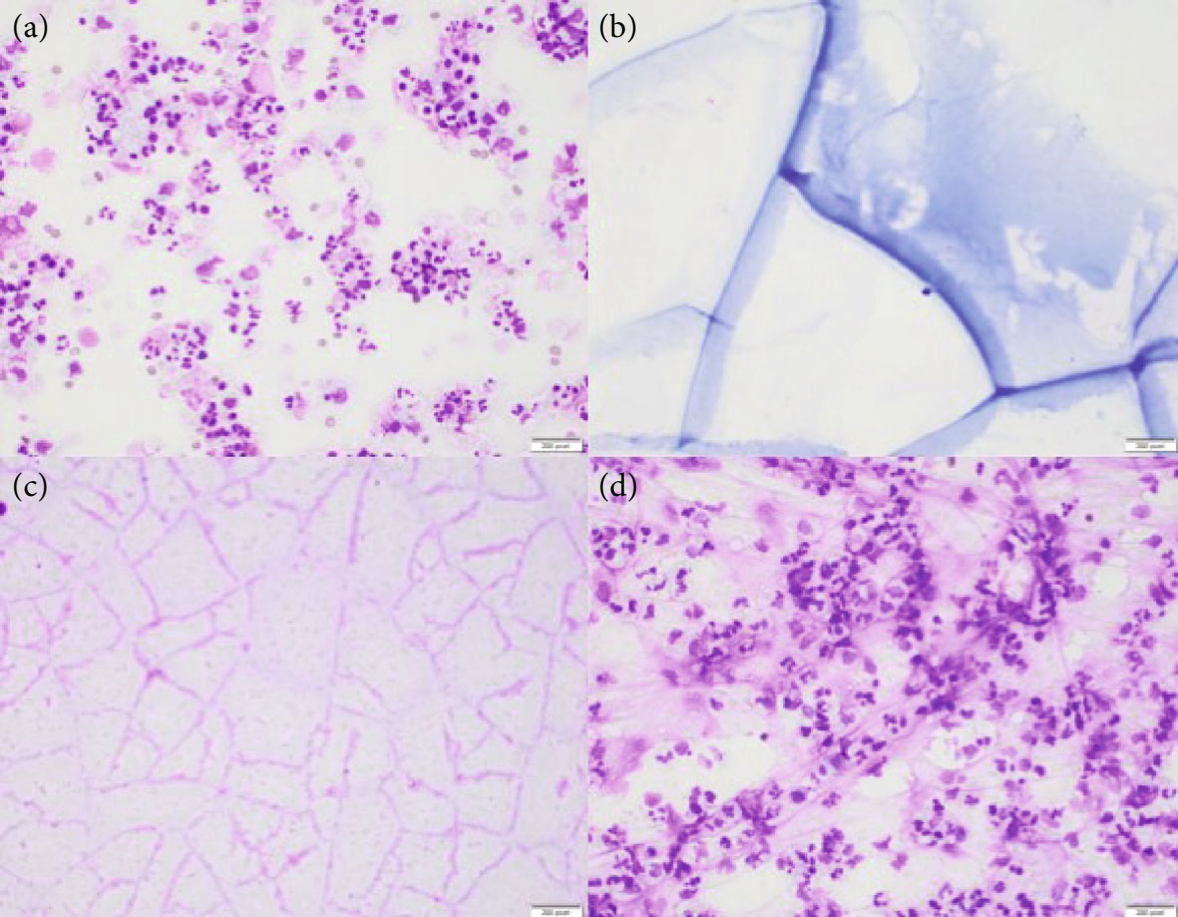
Clockwise from upper left: (a) UPE with discordant cytology smear showing marked cellularity comprised of PMNs, monocytes, and cellular debris in the background. (b) UPE with concordant cytology smear showing proteinaceous, paucicellular fluid with window paning. (c) CPE with discordant cytology smear demonstrating a very similar appearance to (b) with paucicellular, proteinaceous fluid, and (d) CPE with concordant cytology smear showing marked cellularity with admixed PMNs and monocytes and an abundance of fibrinous debris in the background. (Wright Giemsa stains, a–d).

Cytologic samples of concordant and discordant clinical-cytologic diagnoses are shown in Figure 1.

### 3.3. Sensitivity, Specificity, Positive Predictive Value, and Negative Predictive Value with Resolver

A two-step analysis was performed separately using pleural chemistry criteria as the gold standard, with empyema or pleural bacteria as the resolver. Pleural fluid cytology sensitivity, specificity, PVV, and NPV were 78.4%, 95% CI [67.3, 87.1]; 28.6%, 95% CI [17.9, 41.5]; 56.3%, 95% CI [51.4, 61.1]; and 52.9%, 95% CI [38.6, 66.9].

### 3.4. Predictive Clinical Characteristics of Cytology

**Figure 2:**
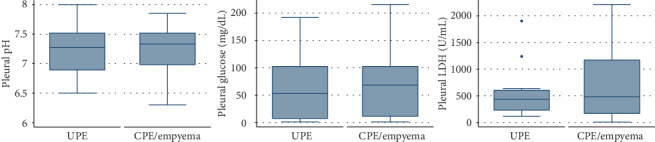
Pleural pH, pleural glucose, and pleural LDH in cytologic diagnosis of UPE and CPE/empyema.

Pleural pH, pleural glucose, and pleural LDH were not significantly different between patients with a cytological diagnosis of UPE or CPE/empyema (Figure 2).

No clinical features were predictive of accuracy, including age, gender, the RAPID score, pleural glucose, pleural bacteria, hemithorax opacity, loculations, or the need for surgical intervention. There was a trend towards the presence of a positive pleural culture (69.8% vs. 42.9%, *p* = 0.07), pleural LDH (2038.9 U/mL vs. 3780 U/mL, *p* = 0.11), and pleural pH (6.97 vs 7.21, *p* = 0.11) in predicting a concordant clinical-cytologic diagnosis of CPE/empyema (Table 2).

**Figure 3:**
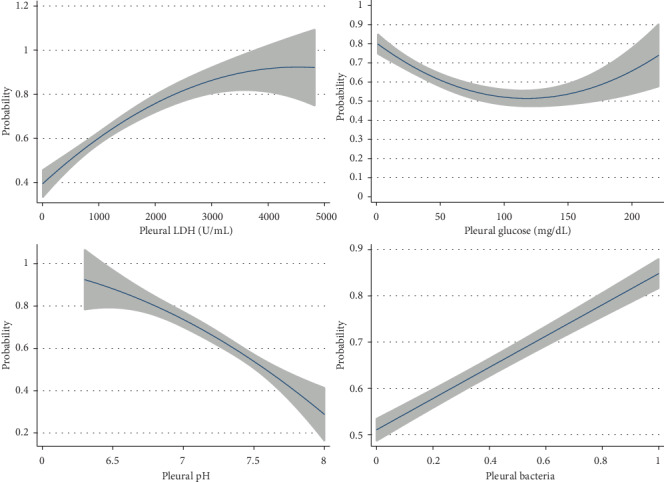
Logistic regression of pleural LDH, pleural glucose, pleural pH, and pleural bacteria.

Logistic regression was performed using pleural LDH, pleural glucose, pleural pH, pleural culture positivity, pleural pus, and cytologic diagnosis. Coefficients were 0.001, 95% CI [-0.0001, 0.001]; 0.006, 95% CI [-0.004, 0.02]; -0.58, 95% CI [-2.39, 1.25]; 1.58, 95% CI [0.28, 2.88]; and 0 and -0.30, 95% CI [-1.18, 0.58], respectively (Figure 3). Only the presence of pleural bacteria was predictive of a concordant clinical-cytologic diagnosis (*p* = 0.02).

## 4. Discussion

Failure to diagnose a CPE associated with pneumonia carries negative consequences such as the need for surgical intervention or death [1]. Therefore, a favorable diagnostic test should possess a high positive predictive value of capturing the diagnosis of CPE. Pleural fluid cytology meets this requirement showing here a PPV of diagnosing a CPE greater than 83%. The NPV of pleural cytology was poor however, less than 40%, and could lead to a pleural drainage procedure when not clinically necessary. Tube thoracostomy with small caliber catheters is relatively safe though, with minimal negative consequences.

While traditional markers often detect a complicated effusion, the pathologist's assessment of pleural fluid inflammation does not appear to increase recognition of an undiagnosed CPE. Of the 42 clinical UPEs that were diagnosed by cytology as a CPE/empyema, only five had poor outcomes due to a lack of drainage. Three patients died due to causes unrelated to the PPE (two from cancer, one from respiratory arrest). Two patients underwent eventual surgical decortication (although one was initially managed with tube thoracostomy, while the other approached CPE criteria with a pleural pH of 7.24). Thus, the addition of pleural cytology is unlikely to detect any undiagnosed CPEs by pleural chemistry.

The presence of pleural space bacteria was a significant predictor of success of the pathologist in correctly predicting a CPE. Inflammatory sequelae including a reduced pleural pH and elevated pleural LDH demonstrated a trend towards clinical-cytologic diagnostic concordance. No other clinical features, including the presence of pleural fluid loculations, the size of effusion, or the severity of illness by the RAPID score accurately determined success or failure of a pathologist to predict presence of a CPE.

There were limitations to this study. First, there is uncertainty of the accuracy of the clinical markers of a CPE [15]. Pleural fluid determinants of a complicated effusion may overdiagnose a CPE. Many parapneumonic effusions diagnosed as complicated will indeed resolve without drainage and are thus considered to be uncomplicated [3]. This affects the predictive values of cytology in the absence of a gold standard. Due to the uncertain accuracy of pleural chemistry, a resolver was utilized as either a positive culture or empyema, features that possess high specificity. Utilization of a two-step approach did not improve the positive predictive value of pleural fluid cytology in diagnosing CPE or empyema. Since many parapneumonic effusions are overdiagnosed by pleural chemistry, the actual PPV of pleural cytology may be even lower than calculated. Second, several of the fluid samples were not tested for LDH. This may have led to misclassification of complicated effusions by LDH as uncomplicated effusions. Of the pleural samples not meeting complicated effusion pH criteria, 21.1% possessed an LDH greater than 1000 U/mL.

The higher degree of cellularity in pleural effusions was expected to be associated with CPEs, although an association could not be confirmed. In fact, a post hoc review of the misclassified specimens did not determine any explanation of the discrepancy in pathologic classification. We originally hypothesized that the intensity of inflammation and presence of a fibrinous exudate were suggestive of loculation formation, but the PPV and NPV of the presence of loculations were poor at 32.0% and 64.7%, respectively. A high degree of inflammation on cytology had, as suspected, an excellent NPV for empyema (94.9%) and NPV for the presence of pleural bacteria (88.2%).

## 5. Conclusion

Pleural cytology does not add an additional value over routine pleural studies in differentiating a UPE from a CPE. It does have a high negative predictive value in ruling out empyema or the presence of pleural fluid bacteria.

## Figures and Tables

**Table 1:** Interobserver agreement. Rows represent Pathologist A, with columns representing Pathologist B.

	UPE	CPE/empyema
UPE	33	4
CPE/empyema	10	90

**Table 2:** Pathology agreement with clinical markers. Values are expressed as mean (SD) for normally distributed variables and mean (interquartile range) for nonnormally distributed variables.

	UPE			CPE/empyema		
	Clinical-pathologic agreement (*N* = 18)	Clinical-pathologic disagreement (*N* = 42)	*p*	Clinical-pathologic agreement (*N* = 60)	Clinical-pathologic disagreement (*N* = 17)	*p*
Pleural LDH (U/mL)	327.57 [141, 574]	307.24 [100, 443]	0.73	2039.94 [487, 2947]	3780 [640, 2215]	0.11
Pleural glucose (mg/dL)	89.31 [75, 109]	100.63 [80, 110.5]	0.28	32.79 [1, 43]	56.5 [1, 40]	0.25
Pleural pH	7.49 [7.41, 7.61]	7.47 [7.36, 7.56]	0.58	6.97 [6.82, 7.20]	7.21 [6.90, 7.35]	0.11
Pleural culture positive (%)	—-	—-	—-	69.8 (46.5)	42.9 (51.3)	0.07
Pleural pus (%)	—-	—-	—-	44.2 (7.6)	28.6 (12.1)	0.30

## Data Availability

The data used to support the findings of this study are included within the article. Individual subject data is available upon request through John Ferguson (jferguson@rockymtnpulmonary.com) for researchers who meet the criteria for access to confidential data.
